# Evaluating fidelity of community health worker roles in malaria prevention and control programs in Livingstone District, Zambia-A bottleneck analysis

**DOI:** 10.1186/s12913-020-05458-1

**Published:** 2020-07-02

**Authors:** Helen Mwiinga Chipukuma, Hikabasa Halwiindi, Joseph Mumba Zulu, Steven Chifundo Azizi, Choolwe Jacobs

**Affiliations:** 1 Department of Health Policy and Management, School of Public Health, University of Zambia, P. O. Box 50110, Lusaka, Zambia; 2 Department of Environmental Health, School of Public Health, University of Zambia, P.O. Box 50110, Lusaka, Zambia; 3 Department of Health promotion and Education, School of Public Health, University of Zambia, P.O. Box 50110, Lusaka, Zambia; 4 Department of Epidemiology and Biostatistics, School of Public Health, University of Zambia, P. O. Box 50110, Lusaka, Zambia

**Keywords:** Community health worker, Community malaria agent, Performance, Evaluation, Malaria, Assessment, Implementation, Fidelity, Quality, Policy

## Abstract

**Background:**

Community Health Workers (CHWs) are an important human resource in improving community malaria intervention coverages and success in reducing malaria incidence has been attributed to them. However, despite this attribution, malaria resurgence cases have been reported in various countries including Zambia. This study aims to evaluate the implementation fidelity of CHW roles in malaria prevention and control programs in Livingstone through performance and service quality assessment.

**Methods:**

A mixed method concurrent cross-sectional study based on quantitative and qualitative approaches was used to evaluate performance and service quality of the CHW roles for selected catchments areas in Livingstone district. For the quantitative approach, (34) CHWs were interviewed and a community survey was also done with 464 community participants. For qualitative approach, two focused group discussions with CHWs and three key informant interviews from the CHW supervisors were done.

**Results:**

Overall implementation fidelity to the CHW roles was low with only 5(14.7%) of the CHWs having good performance and least good quality service while 29 (85.3%) performed poorly with substandard service. About 30% of house-holds reported having experienced malaria cases but CHWs had low coverage in testing with RDT (27%) for malaria index case service response with treatment at 14% coverage and provision of health education at 23%. For other households without malaria cases, only 27% had received malaria health education and 15% were screened for malaria. However, ITN distribution, sensitization for IRS were among other CHW services received by the community but were not documented in CHW registers for evaluation. Factors that shaped fidelity were being married, record for reports, supervision, and work experience as significant factors associated with performance. Lack of supplies, insufficient remuneration and lack of ownership by the supervising district were reported to hinder ideal implementation of the CHW strategy.

**Conclusion:**

Fidelity to the malaria CHW roles was low as performance and quality of service was poor. A systems approach for malaria CHW facilitation considering supervision, stock supply and recruiting more CHWs on a more standardized level of recognition and remuneration would render an effective quality implementation of the CHW roles in malaria.

## Background

Global reports indicate a decline in Malaria incidence by 37% and Malaria mortality rate by 60% between 2000 and 2015 [1, 2]. This has been in part been attributed to Community interventions through Community Health Workers (CHWs) who are a link between the community and the health facility [2–4]. However, the threat of resurgent Malaria is present in various countries including Zambia, Madagascar and Cameroon because of weakening of the Malaria control programs that has been linked to lack of funding, complacency with the malaria situation coupled with poor malaria program execution, purposeful cessation of control activities and lack of cooperation from the community. Great awareness of the threat of resurgent malaria and the development of systems to minimize the decline in malaria incidence and mortality are key to further progress in malaria control [5].

The World Health Organization (WHO) has proposed three main pillars that, if implemented would to move efforts towards elimination of Malaria. These pillar are; to ensure universal access to malaria prevention, diagnosis and treatment; to accelerate efforts towards elimination of Malaria and attainment of Malaria-free status, and; to transform malaria surveillance into a core intervention [2, 3]. These pillars can be best achieved through the primary health care (PHC) strategy that most countries adopted as policy after the 1978 Declaration of Alma-Ata. Community Health Workers are key actors in PHC as links to the community members, and their involvement has proved to be a good initiative in Malaria control activities [4, 6, 7].

Community Health Workers are men and women chosen by the community, and trained to deal with individual and community health problems, and working closely with the formal health care system [2, 8, 9]. Community Health Worker is a general term used for all health volunteers, assisting in community health regardless of a type of disease. However, different names are used in different countries but for the purpose of literature search in the document, the term Community health worker was dominant in the search engines and as such the term was used predominantly. However, national wide CHWs who deal with malaria issues are called Community Malaria Agents (CMA). Over the past decades, studies have shown that CHWs can help reduce morbidity and mortality in settings that have traditionally lacked access to health care [7, 10, 11]. Crucial to sustainable success of CHW programs is strengthening the health system capacity to support with commodity supply, supervision, and appropriate treatment of referred [2, 4, 12]. The CHW strategy, as adopted by most countries in relation to helping improve intervention coverages has proved to be a good strategy in Malaria control activities [6]. However, despite the attribution of reduction in incidence of morbidity and mortality, malaria resurgence cases have been reported in various countries including Zambia thus raising several questions on the implementation of CHW programs [5].

In Zambia, malaria continues to be a public health problem, a major cause of morbidity and mortality in the country, resulting in approximately 6 million cases and 2000 deaths despite substantial progress made over the past decade (HMIS, 2016). Efforts to control malaria are currently being scaled up through coordinated efforts in community interventions such as Indoor residual spraying in the communities (IRS), distribution of long lasting Insecticide treated nets (LLIN), Larval control, Intermittent presumptive treatment of malaria in pregnancy, Diagnostic testing and Malaria case management [13]. These activities were implemented through a phased roll-out in selected districts in the Southern Province of Zambia starting with Choma and Livingstone districts, with the goal of improving surveillance and interrupting transmission [14]. This is because Southern province had made a substantial decline in malaria and is in the low transmission zone (Zone II) [15]. Livingstone district of Zambia however, has had a steady rise in the cases of malaria from 3.5/100,000 population in 2009 to 10.6/100,000 population in 2014 (LDHIS, 2014). Factors contributing to these observations are not well known. It is not known whether this situation is due to the weakening of malaria control programs at the community level or could be partly due to a decrease in community acceptance or participation in malaria programs [5].

In 2013, the Zambia government adopted a Community health worker strategy for early diagnosis and treatment of uncomplicated malaria to achieve malaria elimination in the low transmission zones (zone II) [16]. According to the Zambia Malaria Operational Plan FY 2017, the CHWs in the ICCM approach are provided with diagnostic tools and medicines for the management of common childhood illnesses including the treatment of uncomplicated malaria. Their roles in the management of uncomplicated malaria include; Carrying out diagnoses according to their training and recognizing danger signs, Using RDTs in all cases of fever to confirm malaria before treatment, administering the first-line medicine, referring to the next level and administer pre-referral treatment when danger signs are recognized, instituting measures to reduce body temperature, following up with patients, particularly children under 5 years of age, providing education to the community on the need for compliance to treatment, recognition of danger signs, and prevention of malaria in terms of environmental management, ITN distribution and inspection of ITN use and dissemination of preventive messages such as IRS for instance, advising when to return if the condition persists, and reporting to the facility in their catchment area.

Understanding fidelity and factors shaping CHWs performance in relation to adhering to the CHW components or roles is important for a positive implementation outcome [5]. Periodic performance assessments through surveys and prompt feedback of results on the implementation of interventions to stakeholders in the locality may help to improve malaria control in malaria-endemic countries [17, 18]. Livingstone district is implementing the CHW strategy using voluntary CHWs in Libuyu and Nakatindi communities. The aim of the study was to evaluate the implementation fidelity of CHW roles in malaria prevention and control programs in Livingstone, Zambia, despite the attribution to decline of malaria incidence of morbidity and mortality to CHWs.

The evaluation of the CHW fidelity to roles was based on a framework by Carroll and co-workers. Components of the framework are the intervention, in this case the CHW worker intervention and potential moderators such as facilitation strategies to implementation of the CHW malaria strategy, quality of service delivery and participant responsiveness. Adherence to roles as a measure of fidelity was evaluated through CHW role performance coverage [16] which are Health Education of malaria prevention and control, Community testing with RDT, treatment with ACT for uncomplicated malaria and reporting [19], and also and quality of execution of malaria activities in terms of whether they carry out their roles as expected according to training.

## Methods

### Study design

The study employed a concurrent mixed methods design. The quantitative design used a cross-sectional approach while the qualitative component used a case study. The qualitative study informed quantitative findings hence in the results and discussion, the two methods are mixed in presentation.

### Research setting

This study was conducted in Livingstone districts of Zambia, a tourist capital involved mostly in hospitality industry. About a third of the Livingstone population lives in poverty. Despite others being in formal employment, majority of people are involved in informal employment such as small – scale businesses, cross border trading and fish mongering. The district was chosen based on the fact that despite having moved towards the elimination of malaria, the district currently experiencing resurgent malaria threats (LDHIS 2015) hence the need of assessing extent of fidelity to CHW roles. The study was conducted in the catchment areas for two health facilities - Libuyu urban health facility with a catchment population of 18,057 and Nakatindi health facility with a population of 4925 according to central statistics Office in 2015. Libuyu records the highest malaria cases in the district and both facilities are the only clinic catchments (Nakatindi and Libuyu) where there were active CHWs for malaria prevention and control programs.

### Study population

The study participants for the quantitative component consisted of all CHWs in the two clinics participating in malaria community health activities. Community members were also included as end users through a household survey and because they are key to the measurement of CHW performance in validating or qualifying the performance measure of CHWs through the services rendered by CHWs in the community.

The qualitative study participants included key informants - the program implementers such as the district malaria program officer and facility malaria community focal persons who were interviewed on strategies that affected implementation of the CHW strategy. In addition 10 CHWs from each of the two study sites participated in two FGDs to discuss perspectives on responsiveness.

### Sample size and sampling

For the community survey, the catchment areas were purposively sampled as they are areas were the community malaria agents are found. All the zones in each catchment area were selected (complete enumeration) as they have representation of a community malaria agent in each zone.

Community survey sample size was calculated using the single proportion formula **n**$=\frac{{\boldsymbol{z}}^{\mathbf{2}}\boldsymbol{p}\left(\mathbf{1}-\boldsymbol{p}\right)}{{\boldsymbol{e}}^{\mathbf{2}}}$**=**$\frac{{\mathbf{1.96}}^{\mathbf{2}}\times \mathbf{0.5}\times \mathbf{0.5}}{{\mathbf{0.05}}^{\mathbf{2}}}$**=** 384 + 20% (77) non-response rate: *n* = 463.

The proportion 0.5 was used since the prevalence of a CHW strategy service was unknown. The study sample size of 463 was apportioned to the two catchment areas using probability proportion to size as follows: (4100/11,387)*463 which gave Libuyu 167 Households and (7287/11,387)*463 which gave Nakatindi 296 Households. An equal number of households were selected from each zone. The household head from the selected households, above 18 years of age, male or female were interviewed.

The key informants (KI) for the qualitative study (District Malaria focal point person and the two malaria focal point persons at the two clinics) were purposively sampled. For the two FGDs, 10 CHWs from each study site were purposively sampled from the 40 CHWs. The selection of FGD participants was guided by the CHW facility supervisors.

### Data collection

A checklist was used for collection of quantitative data. Questions for the checklist were based on the conceptual framework for CHW performance [20]. Semi-structured interviews were used in the household survey.

Questionnaires administered to the individual CHWs were adopted from studies that used validated questionnaires using Cronbach’s reliability alpha [20, 21]. The independent variables included age, sex, level of education, marital status, monthly income or incentives, number of household members (those with more than five members tend to have more support at home), number of children under 5 (those with no child under 5 tend to be more active), knowledge in the CHWs understanding of malaria, reason for being CHW, duration of being CHW, financing, health systems factors like training, supply of commodities, and technical support supervision. For the survey, data was collected from sampled households using a semi-structured interview guide to assess coverage of CHW strategy in the community. Household heads above 18 years were interviewed. The survey questionnaire was administered by the trained research assistants. Pretesting of the tool was done manually with a questionnaire in both study sites which allowed for modification of the data collection tool.

For qualitative study, interview guides that were developed based on the implementation fidelity framework by Carroll and co-workers [19] were administered to the CHWs and the KIs (Additional file 1).

### Performance assessment

This is the accomplishment of CHW tasks in Malaria prevention and control against given roles and targets in a given period considering how many CHWs were adherent to the roles. The dependent variable was the level of each CHW’s performance which was developed using five indicators: monthly reporting rate within the previous 6 months, malaria knowledge, health education or sensitizations done, percentage of people tested from the expected target of 40 within 140 m radius of the index case [14], percentage treatment of positive cases by the CHW in their zones. Each indicator was categorized into quintiles (0–4) to standardize the scores making a total of 20 points as highest integrated score. Knowledge of malaria epidemiology and vector ecology, was measured by the respondents’ correct answers on items related to service quality and actions on malaria. There was no prior assessment done to test reliability of the tool but the indicators picked to assess performance was based on a previous study that was published [20] but all the indicators had equal weighting. Reference was made to the reports submitted to the Health center by the CHWs (Table 1). Malaria transmission is throughout the year but the denominator for calculating percentage coverage for performance sorely depended on what the records indicated during the previous 6 months period under review, of which part of peak periods October to December (hot-rainy season was assessed [20].

**Table 1:** Demographic characteristic of CHWs

**Score**	**No. monthly reports in 6 months (0–6)**	**Malaria knowledge**	**Health education**	**% people tested 0–32 and above (0–100%)**	**% malaria positive treated (0–100%)**
0	no monthly reports submitted	lowest (0 score)	none	less than 20,	less than 20
1	1 or 2 submitted	low (1–4)	1 or 2 sessions,	20–39,	20–39
2	3 submitted,	middle (5–8)	3	40–59,	40–59
3	4 submitted,	high (9–12)	4 sensitization	60–79	60–79
4	5 or full reports submitted	Highest (13–16).	5 and above	80–100	80–100

### Quality assessment

Assessing quality of CHW services was done by quantifying CHWs who always carried out active detection, diagnosis and treatment, prescription of anti-malarial, follow-up of patients, and dissemination of preventive measures; as a fidelity measure to community malaria activities with scores given that was used in the previous study [21]. Each item was detailed in how each activity is done in terms of regularity or frequency categorized as “always” = 2, “sometimes” = 1 or “never” = 0. The score for each of the five items was calculated as [total points divided by maximum points] so that each item is given a maximum of one point. A CHW was said to be offering good quality service if they scored at least 4 and above representing at least 80% of the CHW activities always done by CHWs (Table 2). For the variable of follow up of index cases, the assessment was done with an index case present in the register. All the CHW assessed had malaria positive cases that needed follow up.

**Table 2:** Quality Measure

**Index**	**No. of items**	**Highest score**	**Item**
Service quality	5	5	Active detection
			Diagnosis and treatment
			Prescription of anti-malarial
			Follow up
			preventive measures

### Data analysis and management

Quantitative data entry was done using Epi info, and analysis using STATA version 13. In order to identify factors associated with performance for responsiveness and quality of CHWs’ service delivery, Chi square fishers exact test was done to determine whether there was significance of association. The quality of data was assessed by comparing data in the community registers at the health facility level and numbers provided in the reports at the district health office. Data was also checked for completeness and consistency. Further, only data that was complete was included for analysis.

For performance measurement, a chi square fishers exact test was run as performance levels was at two levels (poor< 12 and good 12+). For quality assessment, a chi square fishers exact was used to measure association between the predictor and dependent variables (< 4-less than 80% substandard and ≥ 4–80% + good). Each indicator of the five CHW roles were analyzed based on CHW who always carried out the expected activities for that particular indicator and categorized as substandard if scored less than 0.8 for each indicator and at least good quality if scored more than 0.8 for an indicator. Good coverage is at least 80% achievement according to WHO guidelines.

Qualitative data was managed using n-vivo 10 software after verbatim transcription of all the recordings. Data was analyzed using thematic analysis. This approach allows for categorizing of data into themes so as to identify patterns and trends. Preliminary reading of transcripts allowed for development of a code-list that was imported into the software for coding. Code reports from the coding activity allowed for analysis and interpretation of results.

### Ethics

Ethical approval to conduct the study was obtained from the University of Zambia Biomedical Research Ethics Committee IRB00001131 Of IORG0000777. Authority to conduct the study was also obtained from the Ministry of Health-Livingstone District Medical office. During the data collection process, written informed consent was obtained from the study participants.

## Results

### CHW demographic characteristics

A total of 34 CHWs participated in the study. Nakatindi had 19 (56%) CHWs while Libuyu had 15 (44%). Most CHWs were females (71%) while 29% were males. The age range was between 22 and 62 years with a mean age of 23 years. CHWs age was categorized based on quartiles and the bench march for two lower quartiles was below 40 years and two upper quartiles above 40 years of which 18% were under 40 and 62% were above 40 years old. About 71% had a secondary school education while 3% had no formal education. Only half (50%) of the participants were married. About 47% of them only did some piece work for survival and earned less than K500 (US 53) only on monthly basis while 53% were involved in diverse businesses. A total of 12 (35%) had more than six household members, and 17 (50%) had under five children in their households. Also 17 (50%) had more than 1 year of working as a community malaria agent. Table 3 provides a summary of the background characteristics of the participants.

**Table 3:** Demographic characteristic of CHWs

**Characteristic**	**% CHW responses**	
	**N 34**	
	**n**	**(%)**
**Catchment**		
Nakatindi	19	56
Libuyu	15	44
**Sex**		
male	10	29
female	24	71
**Age**		
< 40	6	18
40+	28	82
**Education**		
no formal	1	3
/primary	8	23
Secondary/	24	71
College/university	1	3
**Marital status**		
Single	17	50
married	17	50
**Occupation**		
Business	18	53
Piece works	16	47
**Income**		
< K500	16	47
K500-K1500	18	52
**Household members**		
< 6	12	35
> 6	22	64
**Number of < 5 yrs children in household**		
None	17	50
1+	17	50
**Work experience**		
6–12 months	17	50
More than 1 year	17	50

### CHW roles and performance

Assessment of CHW roles for the previous 6 months according to records showed that 73% of CHW had good performance in report submission as they had submitted at least four (4) reports from the expected six (6) reports. Only 44% of the CHW managed to give more than 13 sessions of health education in malaria in the community from the expected 19 health education schedules. For index testing, according to the 2013 Malaria training protocols used at the time, Community health workers for malaria were expected to test at least 40 people in households that are within the radius of 140 m [14] and only 29% of CHWs were able to test at least 80% (32) of the expected target of 40. For treatment, only 15% of CHW were able to treat positive malaria cases. However, 97% of CHWs were knowledgeable about malaria by scoring at least 9 out of 16 questions and yet did not perform as expected. The knowledge performance range was from 8 to 13 and the mean knowledge score was 10 with a standard error of 0.22 and a confidence interval of 10.2–11.1. Overall, only 15.7% (5 out of 34) CHWs had good performance in all the CHW malaria roles. The performance scores ranged from 3 to 16 out of the maximum score of 20. The mean performance score was 11 with a standard error of 0.67 and a confidence interval of 9.8–12.5 (Table 4).

**Table 4:** Performance level indicators

**Indicator**	**Total Score**	**Poor (0–2)**	**Good (3–4)**
**Reports**	4	< 4 reports	4+ reports/6
Scores		27% (9)	73% (25)
**H/E**	4	< 13 sessions	13 + sessions/19
Scores		55% (19)	44% (15)
**Testing 40 HH/ index case**	4	< 80%	80%+
Scores		71% (19)	29% (10)
**Treatment**	4	< 80%	80%+
Scores		85% (29)	15% (5)
**Knowledge questions 1–16**	4	< 9	9+/16
Scores		3% (1)	97% (33)
**Overall**	**20**	**85.3% (29)**	**14.7% (5)**

### Implementation fidelity to the CHW malaria roles

Fidelity to the CHW roles, measured in terms of quality adherence to program components such as active detection, patient follow up, diagnosis and treatment, dissemination of preventive measures, was generally low as only 7(21%) CHW carried out the CHW program as it was intended while 27 (79%) had substandard quality as they carried out less than 80% of the community malaria activities. Details on the fidelity levels are provided in Table 3.

For the active detection of cases, 23 (68%) reported going in the community and find malaria cases and were able to find out if the patient had recovered. For follow up role, 16 (47%) always followed up patients in the community to monitor the progress of treatment. And only 11 (32%) always disseminated preventive messages. Concerning the use of RDT for diagnosis and treatment, only 9 (26%) of the CHWs always used RDT for diagnosis and treatment as others (74%) reported stock outs for RDT. This therefore meant that those that had stock out of RDT could not give malaria treatment but referred to the facility for assessment as treatment is based on a positive slide. Prescription information was only given sufficiently by only 3(8.8%) of CHWs risking non-adherence to drug dosage guidelines (Table 5).

**Table 5:** Quality measures indicators

**S/N**	**Indicator**	**Score**	**Substandard < 0.80** **n (%)**	**At least good quality 0. 80+** **n (%)**
1	Active detection	1		
	Scores		11 (32.4%)	23 (67.7%)
2	Diagnosis and treatment	1		
	Scores		25 (73.5%)	9 (26.5%)
3	Prescription of anti-malarial	1		
	Scores		31 (91.2%)	3 (8.8%)
4	Follow up	1		
	Scores		18 (52.9%)	16 (47.1%)
5	preventive measures	1		
	Scores		23 (67.6%)	11 (32.4%)
	**Overall**	**5**	**29 (85.3%)**	**5 (14.7%)**

### Community coverage of CHW malaria services

According to guidelines, all malaria index cases are supposed to be followed up by CHWs for screening, including other household members and other households within 140 m radius. CHWs were also to give health education and treating positive cases as a CHW role in community malaria surveillance. Results indicate that out of the 140 households that reported having had positive malaria, only 39 (27%) were tested by CHW and others 101 (72%) from the health facility which indicates high utilization of health facility services by the community. Out of the 140 positive cases, only 33 (24%) reported having been given health education, 20 (14%) were treated by CHW and the others 120 (86%) were treated at the facility. There was low community coverage in terms of health education, testing and treatment in relation to community follow up of index case response by CHWs. From households that did not report malaria, only 49 (15%) of them were screened and 87 (27%) received health education messages from CHWs (Table 6).

**Table 6:** Community Malaria Management by CHWs

**Community malaria services by CHWs**	**Community malaria management**	
	***N*** **= 464**	
	**Malaria case*n*** **= 140 (30%)**	**Non malaria case*n*** **= 324 (70%)**
Health education		
Yes	33 (23.6%)	87 (26.9%)
No	107 (76.4%)	237 (73.2%)
RDT		
Yes	39 (26.6%)	49 (15.1%)
No	101 (72.1%)	275 (84.9%)
Treatment		
Yes	20 (14.3%)	0
No	120 (85.7%)	324 (100%)

Qualitative findings indicated that the number of households to be covered by CHWs (11,367) was high for the small number of CHWs available (36) which made it unrealistic to meet targets. CHWs were overwhelmed with work as they are a few. Sometimes the same CHWs were taken to other zones that did not have the CHW strategy for malaria activities. Multitasking also increased the workload as they were the same people participating in many other projects, especially those that were offering incentives.

“*We need more CHWs to be trained as the area is too big for one CHW to cover a Zone” P1*, FGD2

### Factors affecting fidelity to CHWs roles

Proportions of CHWs who adhered to the CHW roles measured by performance outcomes was low and the service delivery was substandard despite having high knowledge on Malaria. The factors that were associated with CHWs’ fidelity to roles were marital status, work experience, supervision and reporting records. From the qualitative findings, issues of coverage, remuneration, supplies, ownership and capacity building shaped adherence to the CHW roles.

A chi square fishers exact tests showed that being married was significantly associated to performance of CHW roles with a *p* value of 0.015 of which all good performers 5 (29.4%) were among the married while no one from the unmarried had good performance (Table 7). We also found no correlation between age and work experience, marital status and age or marital status and commodity stock. However, CHWs who were married were more likely to have three or more years of experience than those who were single *P* = 0.016 (Table 8). Supervision by professionals at facility (80%) or NGO was significantly associated with performance (*P* = 0.002) as no one supervised by the NHC chairperson had good performance. Supervision was at facility level but was carried out quarterly, by the Anglican Church supporting the malaria program. Supervision is done by checking books, observing tests and having meetings where discussions of challenges are done. Sometimes Community Malaria Agents (CMAs) were supervised when the health facility workers went for the outreach activities or the NHC chairperson upon submission of reports. However the environmental health officer in charge of the CMAs did not manage to constantly supervise the CHWs due to transport logistics and that other professional staff do not supervise them leaving the CMAs demotivated for work.

**Table 7:** Determinants of CHWs performance in the malaria prevention and control interventions at community

**Characteristic**	**% of CHWs and performance score category**		***p*-value: Fishers exact**
	**< 12 (poor)**	**12+ (good)**	
	**n (%)**	**n (%)**	
Education			
No formal and primary	8 (88.9)	1 (11.1)	1.0
Secondary/tertiary	21 (84.0)	4 (16.0)	
Clinic catchment			
Libuyu	12 (80.0)	3 (20.0)	0.634
Nakatindi	17 (89.5)	2 (10.5)	
Age categories			
< 40 years	4 (66.0)	2 (34.0)	0.25
40+ years	25 (89.3)	3 (10.7)	
Occupation			
Business	16 (88.9)	2 (11.1)	0.6
Other	13 (81.2)	3 (18.8)	
Marital status			
Married	12 (70.9)	5 (29.4)	0.015*
Other	17 (100.0)	0 (0.0)	
Income			
< K500	14 (87.5)	2 (12.5)	1.0
K500-K1500	15 (83.3)	3 (16.7)	
Sex			
Male	7 (70.0)	3 (30.0)	0.138
Female	22 (91.7)	2 (8.3)	
No of household members			
< 6	11 (91.6)	1 (8.4)	0.635
6+	18 (81.8)	4 (18.2)	
No of children U-5			
0	15 (88.2)	2 (11.8)	1.0
1+	14 (82.3)	3 (17.7)	
Work experience in malaria			
6-12 months	17 (100.0)	0	0.04*
Above 12 months	12 (70.5)	5 (29.5)	
Reason for being CHW			
Recommended by NHC	21 (84.0)	4 (16.0)	0.73
Interest in malaria	8 (88.9)	1 (11.1)	
Incentive/payment received			
Yes	1 (100.0)	0	1.0
No	28 (84.8)	5 (15.2)	
Supervised			
Never	0	1 (100)	0.303
Every 6 months	2 (100.0)	0	
Every 3 months	16 (84.2)	3 (15.8)	
Monthly	11 (91.7)	1 (8.3)	
Supervisor			
Clinic	1 (20.0)	4 (80.0)	0.002*
NHC Chairman	5 (100.0)	0	
NGO	23 (95.8)	1 (4.2)	
Record for reports			
Register	0	1 (100.0)	0.05*
Phone	2 (66.7)	1 (33.3)	
Paper	3 (75.0)	1 (25.0)	
Book	24 (92.3)	2 (7.7)	

*significant finding

**Table 8:** Distribution of study participants according to age, work experience, marital status and commodity stock out

Characteristic	N	Work experience in years				*P*-value
		2		3+		
		n	(%)	n	(%)	
Age category						1.00
22–39	6	3	17.6	3	17.6	
40+	28	14	82.4	14	82.4	
Marital status						0.016*
Single	17	12	70.6	5	29.4	
Married	17	5	29.4	12	70.6	
		Marital status				
		Single		Married		
		n	(%)	n	(%)	
Age category						0.175
22–39	6	1	5.9	5	29.4	
40+	28	16	94.1	12	70.6	
Commodity stock-out						1.00
No	1	0	0	1	5.9	
Yes	33	17	100	16	94.1	

*significant at *p* < 0.05

CHWs who were married were more likely to have three or more years of experience than those who were single (Table 8)

### Experience and self confidence

Among those with 6–12 months’ work experience no one performed well compared to 5 (29%) that had at least 12 months’ work experience with *p* = 0.04 (Table 7). Most CHWs 29(85%) seemed not to have confidence in the work they did especially in treating. Treatment protocols were at the clinic and this maybe one of the contributing factors to lacking confidence to administer treatment as they had nothing to refer to in terms of treatment according to age categories. Only those that have more than 12 months experience could administer coartem.

*“They administer drugs and they have drugs though there is a challenge. Some are experienced and others are not experienced. They have fear to administer drugs hence they refer to the clinic even if client is positive”. KI 2*

### Inconsistant supplies

Insufficient stock for use was found to be a challenge. This included RDTs, anti-malarial drugs (ACT) and storage equipment like bags for carriage or keeping RDTs and drugs which risks compromising potency if put on direct sunlight. Sometimes when clients with fever were attended to but with a negative result, there was no antipyretic or pain killer but were just referred to the health facility. For ACT, only CHWs from peri-urban areas were given but those near the health facility just collected for clients in order to treat, with instructions on how it should be taken. Lack of thermometers, scales and referral forms was also reported. Insecticide Treated Nets were given but demand was higher than supply. Pregnant women and under five children were prioritized to be given an ITN as each bed space may not have an ITN.

The reporting record was found to be a factor associated with performance where 92% of non-performers and 8% for good performers used books and not standard registers with *p* value =0.05. However, a variety of reporting tools were reported to have been used like phone, paper and book which have no standardized way of recording (Table 7).

*“We who work are free but we have challenges with reagents in some cases. We need RDT consistency for us to continue working, as people are now saying the CHW has stopped testing. People will neglect us, so give us RDT so that we continue working. A long time ago people came because we had access to RDTs but now people come but can’t be tested. Now people come and bounce, we feel bad as we can’t do anything for them as we don’t want to help meanwhile it is just things we lack to use.p4 fgd2*

### Financial resources

Key informants indicated that it was very difficult to achieve the malaria targets without CHW incentives. This affects their performance since the community malaria agents would rather work where there is an offer of an incentive as they even feel the malaria program is lagging behind in terms of incentives compared to other health programs. However, Community health workers indicated that what motivated them to continue working even without incentives was the fact that they were told that they were volunteers from the beginning and so they were self-driven to serve the community and wanted to be part of malaria reduction. Difficulties in mobility affected their performance as they had to travel distant places hence difficulty to cover vast areas.

“*From the beginning, we were told we were volunteers and we understood. We do sacrifice so at some point they should remember us that we have families. We don’t work and at the end of the day we need to see to it that the family has food on the table. The world we live in now is different from the way it was before. Times are different from times back. Let them consider us even just a bit for us to be able to continue”. P1* FDG

### Ownership by the district

The CHWs felt discouraged to work as they felt that the district did not pay much attention to them. They however received support from the Anglican Church which offered support in terms of training the CHWs and provision of mosquito nets for distribution, ITN use inspection and health education. The district only come in if asked especially if there were leakages they help talk to the city council who also supports by ensuring water leakages are controlled as children play in the stagnated water.

*“Let the district also plan for us besides the Anglican. They should claim ownership and have their own program. They should also provide refresher courses. After all the Anglican found us at a clinic for the district. Like the days of old they should give us refresher course, they should not relax, not waiting for Anglican who get from other donors and us the church.” P3 FGD1.*

### Recognition and community connectedness

The CHWs are known in the community and therefore needed some formal recognition with certificates of training in the work they did including the similar identification with identity cards and aprons. This enthusiasm for recognition made them want to work at the center instead of working from the community and have to constantly remind them to go work in the community as the health center was just for them to bring reports. This may be the reason for poor community coverage with regards to their community malaria roles.

*“We need certificates of training and also identity cards, and refresher courses to have latest updates so that we are not left too much behind. We just have apron and T- Shirts of which others still do not have, especially in rainy season, we need rain gear you can be protected and the books do not get wet.” P2 P8 FGD2.*

## Discussion

This study evaluated fidelity to the CHW roles for malaria considering the moderating processes as it was intended in terms of content and coverage. Overall, Fidelity was found to be low considering that a lot of factors were affecting the implementation of roles and these were individual CHW factors and a great deal of health system factors. In the qualitative findings, community factors were found to affect fidelity of CHW to their roles in community malaria programs, a finding similar with a systematic review study by Rowe on the effectiveness of strategies to improve health-care provider practices in low-income and middle-income countries which reviewed that training alone for CHWs was not effective but with added community support [22].

### Facilitation strategies shaping fidelity to CHW roles

#### Individual CHW factors

Only 15% of CHW were able to work according to the way the program was intended with diagnosis, treatment, follow up, health education and reporting despite them being knowledgeable unlike in a Cambodian study were VHW were not knowledgeable as a reason for poor quality service [21].

Being married was a significant factor of performance in this study as all good performers were married. This is similar to study were married CHWs gave a higher performance than others [20]. This could be because they have more family members to help with household duties or that they may have an extra source of income from the other partner. Having fewer household duties encourages CHWs to work more actively and reduces the dropout rate as one of the barriers preventing a good CHW performance was a heavy amount of household duties [23] though this was not significant in this study. Recruitment of married CHW for malaria interventions with priority placing may help in sustaining good performance in improving coverages. This however is a hypothesis that should be tested to see if it is valid in this setting.

Work experience was another significant factor that related to CHW performance. This was indicated by the fact that CHWs who were good performers all had worked for more than 1 year. Longer work experience was also a similar finding in this study as confidence is belt more with the longer the period one works [20, 24–26]. The program identified experienced CHW to be receiving zonal reports from the CHWs that had little experience. Longer work experience entails having more opportunity to receive effective training, supervision and any incentives and to build a confidential relationship with community members [25, 27]. Familiarity with the work motivates CHWs to apply for position like that of a Community Health Assistant (CHA), another strategy being rolled out in Zambia [28].

Most CHW were able to conduct diagnosis with RDTs skills that they had acquired through the training programs [29, 30] but prescription messages were poor during follow ups which risks resistance due to non-adherence to treatment. This could also have been due to the issues of confidence to treat, a finding different from a study in Ghana where CHW adequately treated even children with malaria [31]. Occasional onsite quality supervision to actually see what they do is vital in order to ensure to quality service especially that some of them are supervised by the fellow malaria CHW and that the facility supervisor is facing challenges in mobility to enable quality supervision.

CHWs drop-out rate in this setting was reported to be at 5% (2/36) reasons being lack of monitory incentives. Demotivation due to unfunded program for CHWs to make ends meet [32, 33] affected fidelity hence CHWs just stayed in the community or sought other programs that have incentives. Improving non-monetary incentives such as providing them with materials that identify them as community-based health workers e.g. badges, t-shirts, and so on; frequent refresher courses and the exchange visits. To avoid demotivating CHW and health workers alike, sufficient remuneration, supplies of RDT, drugs and ITNs and job aids need to be consistent, including relevant infrastructure and supportive supervision may improve adherence to CHW roles [28, 32, 33]. This may also improve their social prestigious need for recognition in the community and improve community connectedness as indicated in a study by Strachan DL that CHWs value feedback and feeling connected to the health system and their community, are motivated by status and community standing, and want to be provided with the necessary tools to perform [34].

#### Health system factors

##### Governance

CHWs are aware that they are volunteers always willing to work with 97% of them being knowledgeable but this knowledge did not yield good performance or good quality service because the organization system has not put in place adequate necessities for this strategy to work as intended. The main qualitative factors that surrounded performance and fidelity of CHWs to the malaria program included insufficient remuneration, reduced mobility, inadequate quality supervision, inadequate funding, and work overload for CHWs, inconsistent supplies of stocks, poor coordination with partners and lack of initiative for capacity building. CHW performance is hard to achieve and to maintain without sufficient consideration for funding and other motivating factors like transport and remuneration [20, 25, 35, 36].

Supervision was a significant factor associated with CHW performance specifically the supervising organization. The CHWs were being supervised by the existing NGO, the clinic and chairman of the program. Those who had good performance were supervised by the clinic though the facility supervisors faced challenges with mobility for following up CHWs in the field. Supervision not only needs frequency but quality input as high quality supervision is one of the key factors in improving a CHW’s performance [2, 20] as evidence from a systematic review on impact and implementation of supervision suggests that improving supervision quality has a greater impact than increasing frequency of supervision alone with supportive supervision packages, community monitoring and quality improvement/problem-solving approaches, though evaluation of all strategies is weak [37]. For instance in Kalabo study, frequent supervision did not have a positive impact on CHW performance as quality was reported to be poor and almost half of the community health workers do not experience any benefit from the supervision [26]. Supervisors should therefore have adequate health knowledge and conduct routine supervisions to sustain a high performance and responsiveness from the CHWs and they should have standardized method or checklist for the supervision of community health workers. This is because supervisors do not receive specialized training as mentors, but assume that role based on their academic training and orientation to program performance assessment through workshops.

##### Reporting system

Reporting record used was found to be a significant determinant of performance. There is poor CHW program coordination and collaboration with regards to the supporting organization and ownership by the supervising district. A standard register and a reporting tool that is common to both supervising organizations is necessary for a common goal. The stake holders have a direct influence on the health system factors and are to produce guidelines, registers, reporting tools, checklists and evaluation tools [33] though an innovation to improve the information system through use of phones for reporting has been effected [38] without data being captured by the local district. CHW had no standard reporting tools and were expected to submit a report to their zonal chosen CHW supervisor who takes to the chairman of the Malaria CHW program selected among the CHWs. Other CHW activities were not recorded and CHWs concentrated only on diagnosis and treatment, neglecting other CHW roles for malaria prevention and control. This is similar to a study by Yasuoka et al. where Village Health workers (VHW) concentrated only on diagnosis and treatment [21]. Apart from the health education, testing, and treatment from the survey, a lot more services are rendered by CHWs but not reported and these included ITN distribution, ITN utilization inspection, households sprayed after CHW sensitization, referral, burying of trenches. The qualitative results however indicated doubt in the authenticity of CHW reports as they are rarely supervised and their register records are taken as gospel truth. There is therefore need for a standard reporting tool that will cover all the CHW roles capturing all details required as other details were missing in the improvised registers. This may also aid in supervision by the supervising officers visits [26].

##### Service delivery

Fidelity to the malaria CHW roles was poor due to lack of supplies a cause of inactivity of some CHWs in terms of active detection and treatment [26] similar with the implementation challenge of the test and treat intervention in another part of Zambia where it was concluded that with limited resources, coverage and diagnostic tools, reactive screen-and-treat would not likely be sufficient to achieve malaria elimination but with reactive focal drug administration as an alternative strategy [14].

##### Human resource

It is almost impossible for 34 CHWs to cover 11,387 households without inefficiency as CHWs tend perform poorly due to large population coverage and multiple tasks CHWs and get overwhelmed with so many programs [39] . Scaling up in terms of capacity building for more man power with priority placing may foster good performance in the malaria CHW program. Scaling up of these malaria CHW interventions, promoting continued use of CHWs in national programs as an important human resource that contributes to long term impact of interventions [6, 40]. The government endorsed a CHA assistant program to help meet the human resource demands who are to be liaison between community and the health facilities but discussions with CHAs showed that because of the limited number of trained staff at health posts, it was resolved that CHAs should spend more time at the health posts than in the community [28] hence local malaria CHWs who are always present in their communities will continue being an effective strategy to in the elimination of malaria for community surveillance improvement as one of the WHO pillars in malaria elimination.

The strength of the study is that it a mixed methods study which explores both quantitative and qualitative and this gives an in-depth explanation of barriers to implementation fidelity of the malaria CHW program. It also assesses the services received through the community survey and hence is a comprehensive performance and fidelity assessment of CHW intervention in malaria. However, the limitations were lack of a standard measurement tool for an integrated approach for CHW community malaria interventions hence performance measurement and quality assessment indicators were adopted from previous studies. To evaluate CHW’ service quality, only self-reported data were used, and the actual community experiences were not taken into account in terms of utilization of malaria CHW services. The validation of self-reported indices regarding service quality needs improvement. However, possible attempts were made: for instance, self-reported data were double-checked with CHW’ records in their monthly reports; data used to assess performance, submitted to the CHW malaria supervisor regularly. The analysis of association did not take into consideration the confounding variables as multivariate analysis was not done as the sample size for CHW was small. However, multivariate analysis will be done with survey data when analyzing utilization of CHW for malaria interventions.

## Conclusion

The study findings indicate that fidelity to the malaria CHW roles was low in that adherence to the program as it was intended through performance outcome was poor and quality of service was substandard. Findings suggest that CHWs can still adequately contribute to the elimination of malaria in this setting with attention to health system support. A systems approach for malaria CHW facilitation considering supervision, stock supply and recruiting more CHWs on a more standardized level of recognition and remuneration would render an effective quality high implementation fidelity of the CHW malaria roles for this setting.

## Supplementary information

**Additional file 1.**

## Data Availability

The datasets used and/or analysed during the current study available from the corresponding author on reasonable request.

## Abbreviations

ACT: Artemisinin Based Combination Therapy
CHW: Community Health Workers
CMA: Community Malaria Agents
CSO: Central Statistics Office
FDG: Focused Group Discussion
IRS: Indoor Residual Spraying
IPT: Intermittent Presumptive Treatment
LLIN: Long Lasting Insecticide Treated Net
PHC: Primary Health Care
RDT: Rapid Diagnostic Test
LDHIS: Livingstone District Health Information System
HH: House Holds
H/E: Health Education
