# Supplementary material for: Evaluating fidelity of community health worker roles in malaria prevention and control programs in Livingstone District, Zambia-A bottleneck analysis
Source: BMC Health Serv Res. 2020 Jul 2;20:612. doi: 10.1186/s12913-020-05458-1 (PMC7331272; doi:10.1186/s12913-020-05458-1)
Supplement: Supplementary file 1 — Additional file 1. [file 12913_2020_5458_MOESM1_ESM.docx]

**Appendix 1: CHW Focused Group Discussion Guide**

**CHW FOCUSED GROUP DISCUSSION GUIDE**

**FGD Number └─┴─┴─┘└─┴─┴─┘└─┴─┴─┘**

**Location……………………. Facility ……………………… Interviewer code……….**

**Date of FGD………………………**

Malaria cases are increasing in our community and so we are conducting a study on the Performance of CHWs in malaria prevention and control in Livingstone district as community links. We will be asking you different issues about your work as CHWs in Malaria control.

1. Describe your roles in malaria prevention and control? Are they clearly defined to you?
2. Explain your experience in your work as CHW agents for malaria?
3. How do you perceive yourselves as being appropriate for the community malaria work, and how effective do you think you are in the fight against malaria.
4. Kindly provide your views on the following issues with regards to malaria prevention and control.

- support
- supervision
- training
- supplies
- financing

1. What do you think is the cause of the resurgence of malaria in Livingstone District?
2. What challenges do you meet in your work as community health workers?
3. What motivates you to continue working as CHW in malaria programs?
4. What do you recommend should be done to enable you work well.
5. Any other issues?

**We thank you most sincerely for sharing your opinions**

**Appendix 2: Program Implementers Interview Guide Participants Number…………..Organization………………….**

Malaria cases are increasing in Livingstone district as indicated by the District HIMS and so we are conducting a study on the Performance of CHWs in malaria prevention and control in Livingstone district as community links. We will be asking you different issues about Community malaria interventions and CHWs in Malaria control.

The interviews will be strictly confidential and will only take 45 minutes. With your consent I request to start the interview.

**Questions**

1. What do you think are the factors influencing the Performance of CHWs in health services delivery in Livingstone district? (health system and community factors)
2. Describe how the CHW malaria program implemented and coordinated?

- Explain the characteristics CHWs you recruit for malaria interventions?
- Workload-Explain how the CHWs do their work, as delegated or volunteered?
- Do you give them clearly defined of tasks and roles for malaria prevention? Explain their roles?
- How are CHW Selected and recruited? Is the community involved
- What motivates the CHW to continue working?
- How do you supervise supervised the CHW and how do you evaluate the CHWs?
- Training-any refresher courses or trainings you take them to?
- What Protocols and guidelines do they use and any job aids they use?
- Describe the communication process and reporting processes with the CHWs?
- Describe how the supply system is managed to ensure consistently in their work?
- What do you think is the cause of malaria resurgence in Livingstone district?
- How is the performance of CHW? Are they relevant to the system with regards to malaria control programs

**Closing Remarks** Are there other people you think we should talk to concerning the same? Have we covered everything you think is important?

**Debriefing:** Thank you very much for your time. Your knowledge and insights will be very helpful and valuable. When the process is complete, I will be happy to share a summary of the findings. Thank you again
